# The Clinical Implications of the Academic Performance of the Siblings of Individuals With Autism Spectrum Disorder

**DOI:** 10.7759/cureus.29116

**Published:** 2022-09-13

**Authors:** Ruimin Huang, Shanthi Potla, Sushen Bhalla, Yousif Al Qabandi, Savitri A Nandula, Chinmayi S Boddepalli, Sai D Gutlapalli, Vamsi K Lavu, Rana Abdelwahab, Pousette Hamid

**Affiliations:** 1 Neurology, California Institute of Behavioral Neurosciences & Psychology, Fairfield, USA

**Keywords:** bap, relationship, adhd, asd, siblings

## Abstract

We all know that autism spectrum disorder (ASD) can affect academic performance. Many children with autism face different challenges at school. However, less attention is paid to the siblings of autistic children, who are at a high risk of ASD or the broad autism phenotype (BAP). Recent data also shows that many siblings of ASD children suffer from neurodevelopmental disorders, mental health problems as well as poor academic performance. This review will look at the possible etiologies of the poor school performance of autistic children's siblings, with an emphasis on the challenges they face. We will also highlight the clinical implications of these findings, and the possible solutions that can help this vulnerable group.

## Introduction and background

In 1943, Kanner described autism as a unique syndrome found in young children that disrupt their social and emotional relationships [1]. Autism, known as an autism spectrum disorder (ASD), is currently defined as a neurodevelopmental disorder. Its typical symptoms include impairments in social interaction, difficulties in communication, restricted interests, and repetitive behaviors, which can lead to significant social, communication, and behavioral challenges [1]. Most children with ASD suffer from a significant learning disability, except for some high-functioning autism patients [1]. In 2021, about one in 44 kids were diagnosed with ASD in the United States, according to the Centers for Disease Control and Prevention (CDC) report [2], attracting more attention to this topic in medicine. In the past decades, we have done thorough research on ASD children. However, in recent years, the siblings of autistic children have started to receive little attention. Enough evidence indicates that siblings of autistic children are at a higher risk of mental health illnesses and deviant behaviors, which can affect their academic performance [3].

Genetic liability plays an important role in ASD etiology [1]. According to the research, younger siblings of children suffering from ASD can have the disorder as well. The probability is estimated at 20-25 times the average population. Some kids show subclinical symptoms of ASD, which is commonly referred to as the broad autism phenotype (BAP). According to some research, the siblings of autistic children are more vulnerable to BAP [4]. In addition, the complexity of genetic mechanisms involve a variety of clinical expressions and symptoms [1]. There is a possible connection between autism, attention deficit hyperactivity disorder (ADHD), anxiety, and obsessive-compulsive disorder (OCD) [5]. Evidence shows a higher rate of ADHD in the siblings of ASD children [6], which can cause learning difficulties.

Even for unaffected siblings, living with children with ASD can be an experience influencing their later life beyond imagination [7]. Sibling relationships are important in people's lives and have unique implications for individual development and adjustment [8]. Siblings can influence one another directly and indirectly [8]. Research shows that siblings of autistic children easily get angry, embarrassed, frustrated, and upset by the affected children [7]. In addition, they receive little attention from their parents, making the situation worse [8].

This review tries to give a broad overview of the possible causes of the relatively poor academic performance of the siblings of kids with ASD and its clinical implications. In addition, we discuss the relationships between siblings and their autistic siblings, how their learning ability is affected, and the possible solutions to this situation.

## Review

Poor academic performance in autistic children with normal intelligence quotient

According to the national data, 1/3 (31%) of autism spectrum disorder (ASD) children have an intellectual disability, 1/4 (25%) have a borderline intelligence quotient (IQ) (IQ 71-85), and almost half (44%) of the patients have average or above-average IQ [2,9]. Clearly, children with low or borderline IQ scores have relatively difficult experiences in school. The clinical research, however, unveils that most autistic children with average or above-average IQ, such as high-functioning autism, may still struggle academically or get unsatisfied performance relative to their IQ [10]. They demonstrate significant learning difficulties in many domains [11].

Some autistic children with an average or above IQ (IQ ≥100) struggle academically due to the main symptoms of ASD, including poor social communication, narrow interests, and concrete thinking [10]. Besides, some specific challenges, such as organized writing, comprehension, and solving math problems, seem to be experienced by autistic children with average or above-average IQ. Also, executive functioning (EF), like organizational skills, time management skills, initiation, and prioritization, significantly affect academic performance [11]. Around 1/3 to 2/3 of children with high-functioning autism have EF deficits. They show impairment in planning, flexibility, inhibition, generativity, metacognition, and action monitoring [12]. Some patients can not initiate roles or multitask [11]. The ability to use proper learning skills in different situations is also essential [11].

All autistic children encounter flexibility problems regardless of IQ [1]. Furthermore, most of them have abnormal sensory processing and attentional deficits, which cause difficulties in learning [11]. All this explains why autistic students have discrepancies between intellectual ability and academic performance. Clinically, common challenges for high-functioning autistic children include initiating tasks, concentrating, proper planning, managing multitasking, and organizing materials [13].

The connection between ASD and attention-deficit hyperactivity disorder (ADHD) is very common [12]. According to the data, 52-78% of autistic children also have ADHD [14]. Recent research suggests shared genetic underpinnings of ADHD and ASD [12]. It was estimated that 50-72% of genetic factors show a relationship between ASD and ADHD [15]. Individuals with ASD and ADHD showed worse performance on the attention test, such as the continuous performance test (CPT) [16]. They displayed more unstable reaction time, more omission errors, and poorer sustained attention [5]. These also make high-functioning autistic kids struggle academically.

The recurrence risk in siblings of individuals with autism spectrum disorder

The etiology of autism spectrum disorder is heterogeneous, and the main contributors are genetic factors and environmental factors [17]. The precise causal mechanisms remain unclear. Some research studies show that mutated genes in Wnt, sonic hedgehog, and retinoic acid (RA) are related to the etiology of ASD [17], which backs up the theories of genetic contribution. There is a higher recurrence rate in families with two or more autistic children [18].

As early as 1977, a twin study had been done to prove the heritability of autism [19]. Another twin study had shown that if identical twin siblings have autism, 36-95% of their siblings will also have autism, and the chances will be 31% among non-identical twins [20]. In 2016, a meta-analysis reported a heritability range between 64% and 93% [21]. Recent research further displayed an 8.4-fold increase in the risk of ASD in kids with older autistic siblings compared with the controls [22].

**Figure 1:**
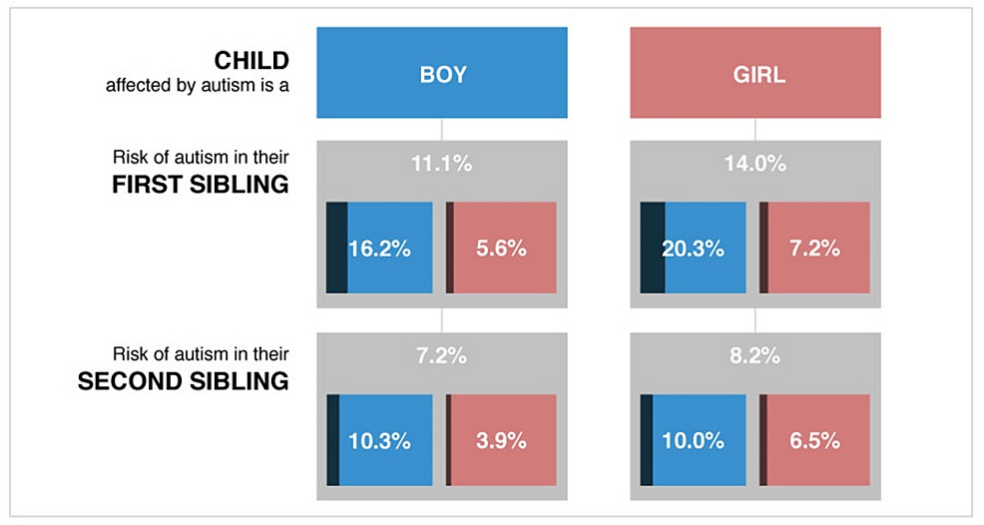
The recurrence rate of autism spectrum disorder in first and second siblings of the affected kids compared with sex Reproduced from https://www.spectrumnews.org [25]

The chance of ASD in kids with autistic siblings is about 20-25 times higher than in the general population [23]. The risk of full siblings is three times that of half-siblings [24]. The possibility of ASD in the younger siblings of autistic kids is also high. On average, around 10-20% of younger siblings of autistic children will be diagnosed with ASD later in their lives. Males are more affected than females [22], as shown in Figure 1 [25].

Broader autism phenotype in the siblings of autistic patients

The recurrence rate of traditional autism spectrum disorder (ASD) in the siblings of autistic children is high. Physicians also noticed that an additional 20% of siblings who have not been diagnosed with ASD had a history of language delay. Among them, about 50% exhibited autistic qualities of speech [23]. Researchers used the Social Responsiveness Scale to test, and the result supported that the chances of subclinical autistic traits are higher in kids with more than one family member with ASD [26]. Characteristics traits similar to autism but do not meet diagnostic criteria of autism spectrum disorder are referred to as broader autism phenotype (BAP) [27]. BAP is not a new concept. As early as 1944, Kanner discovered some common traits shared by the parents and their autistic children, which introduced the concept of broader autism phenotype (BAP), also known as subthreshold autistic traits [28]. In the past decades, more and more siblings of individuals with ASD have been diagnosed with BAP. Most studies focused on the patients themselves, and there was minimal attention paid to the siblings. In 2011, Ozonoff et al. studied 600 children in the USA and Canada and found that at least one person showed BAP symptoms in half of the families with ASD [23]. Another study involving 719 high-risk siblings suggested that about 1/4 of them would show signs of BAP by the age of three [29]. The atypical behavioral signs, which include social-communication impairment, cognitive deficits, and internalizing problems, were shown as early as 12 months, but they were less severe compared to those of ASD [30]. So far, two subgroups of BAP have been identified by the Baby Siblings Research Consortium (BSRC). The first group has more prominent autistic symptoms (still subthreshold) but without language or cognitive delays. In contrast, the second group has notable language and cognitive delays but relatively fewer autistic symptoms [31].

The large percentage of shared genes and environmental factors shared by biological siblings attract the scientists' attention [18]. With more studies about siblings being done, researchers found that the rates of BAP are much higher among siblings of autistic individuals than in controls [23].

Scientists tried to find the etiology of BAP via the genetic mechanism of ASD. As the symptoms and severity of ASD vary because of the different possible expressions of genes [32], some researchers believe that BAP might be a varied expression of autism-specific traits [9]. Studies showed that most of them, including those grouped as 'normal' later in their lives, experienced problems with disengaging attention in their early lives, and they also exhibited difficulty with executive functions (EF) [33].

The signs of BAP are generally recognized as deficits in social functioning, restricted but repetitive behaviors and interests, pragmatic language difficulties, cognitive deficits, and authoritarian personality [23]. They also show reduced efficiency in planning and attention shifting and lower levels of emotion recognition tasks [34]. Deficits in social skills development are also shown in some studies, especially in communication and language difficulties [35]. It is almost similar to ASD but with less severity [27]. Individuals suffering from BAP can still function in society, although with some aspects of deficits. So far, a precise diagnostic conceptualization of ASD still is yet to be established. Hence, the exact characteristics of BAP are still controversial [23].

The concept of BAP inspired scientists with different theories of the inherited pattern of ASD [23]. Scientists noticed that the siblings of individuals with ASD are also at a high risk of being diagnosed with other disorders, such as attention-deficit hyperactivity disorder (ADHD), delayed language development, dyslexia, oppositional defiant disorder (ODD), and anxiety disorders [23]. Iverson et al. found that siblings of individuals with ASD had an attenuated range of arm activity changes when reduplicated babble onset and showed delayed language development at eighteen months. Most of them seem to have different degrees of impaired vocal-motor behaviors in the first few years of their lives [36]. Hudry et al. noticed lower levels of receptive vocabulary in most siblings of autistic children by fourteen months [37]. Moreover, quite a few were diagnosed with other disorders, such as delayed language development, during the study [23]. And the siblings of autistic kids, who have shared the most gene with their affected siblings, are the most vulnerable [18]. Not all persons affected by the pervasive cognitive deficit have challenges in their daily life; some might have mild symptoms [38]. If the symptoms worsen over time, they can turn into BAP or autism [23]. Therefore, Folstein and Rutter hypothesize that a pervasive cognitive deficit existing in children with ASD and their families is inherited, not autism itself [39]. For example, there have been numerous studies of siblings of autistic kids showing slow development of the brain, central coherence, executive function, recognition of facial emotions, and face processing [40]. Hughes et al. found a higher chance of siblings of autistic children having deficits in set-shifting [41]. Scientists noticed, as early as 36 months, that siblings of autistic children showed pragmatic language problems [42].

What is the academic performance of the kids with BAP? Fombonne et al. identified that the group with BAP had a significantly lower intelligence quotient (IQ) than the control group [43]. Chuthapisith et al. had concluded similar results [44]. Besides, relatively poorer efficiency in verbal fluency, planning, attention shifting, and executive function also affect the ability to study in an academic setting [41]. The siblings with BAP exhibit variant deficits in motor and communicative development; some are permanent, and others are transient. Studies showed that deficits in the motor and communication of infants would have negative effects on their development [23]. For instance, delay and limited interactions with their physical and social environments would fundamentally alter the response from environments to the child [45]. This may lead to reduced shared topics with the communication partners, affecting the child's frequency and nature of linguistic input in turn [46].

Moreover, a delayed exhibition of the child's learning ability will give a false impression of the child's developmental level to the teachers; therefore, the feedback provided by the teachers is not optimal for learning [46]. However, we need to point out that standardized criteria for BAP have not been established, making the studies in this area more complicated [23]. The highly diverse design, participant groups, the number and ages of participants, and instruments used, make the findings hard to integrate. More standard research is needed.

High risk of attention deficit hyperactivity disorder in siblings of autistic individuals

As we have mentioned, the siblings of autistic individuals have a higher rate of getting autism spectrum disorder or broad autism phenotype. How about the siblings without these two? They usually get less attention from their parents than their siblings with ASD [7]. For the past half-century, most of the studies focused on ASD children. Even though more focus was put on the high-risk siblings, those who display early signs of developmental delay or variant autistic traits still attracted more attention from parents and physicians. However, more and more parents have been concerned for their unaffected kids in recent decades [7]. Studies on the siblings of individuals with ASD are still in an early stage. There are still many unknown facts. We tried to collect the information available so far to look deeper into their development and its effect on their academic performance.

During clinical practice in the last century, physicians noticed a familial co-aggregation pattern of ASD and attention deficit hyperactivity disorder [47]. Research done in Sweden, studying 1,899,654 individuals born between 1987 to 2006, showed that relatives of autistic kids were at higher risk of ADHD than the general population. Monozygotic twins have a stronger association than dizygotic twins and full siblings. There seems to be an association between ASD and ADHD [48]. Another study involving 6022 siblings of 3578 ASD children and 22,127 siblings of 11,775 matched controls concluded a higher chance of getting ADHD in siblings of ASD cases [49]. A twin study showed that the probability of the monozygotic co-twins of individuals with ASD diagnosed with ADHD was 44%. In comparison, the number of dizygotic co-twins is 15%. Furthermore, the chance for the full siblings of autistic children having ADHD is 3.5 times that of the general population [47]. We are trying to find the connection between ASD and ADHD from the etiology perspective. Scientists studied the genetic mechanism, and the result showed that 50-72% overlap genetic factors between ASD and ADHD [50]. In addition, a binomial test has been used to evaluate the overlapping of the linked regions. There was significant evidence of linkage overlap for ASD and ADHD in seven chromosomal regions [51]. Imagine-study displayed that reduced corpus callosum volume and left the frontal grey matter in both diseases [52]. These explain the overlap of ASD and ADHD in the same patients and the familial co-aggregation pattern of these two diseases. Another study found that siblings of high-functioning autism are more prone to ADHD. In contrast, those with low-functioning autism are more prone to having oppositional defiant disorder (ODD). Furthermore, unaffected siblings of high-functioning autism have relatively more severe ADHD-related symptoms than the low-functioning and control groups [53].

ADHD is commonly recognized as an externalizing problem in childhood and adolescence and is associated with poor academic performance in the international literature [16]; while oppositional defiant disorder (ODD) is defined as defiant, uncooperative, and hostile behavior toward peers, teachers, parents, and even authority [54]. ODD is a common comorbidity of ADHD. ADHD and ODD play an essential factor in mental health and academic achievement [55].

Risk of other development challenges in the siblings of autistic children

A study reported that about 30% of unaffected siblings showed retarded growth in three years old kids, and up to 43% had impairments across a handful of domains which raised clinical concerns such as language and motor [56]. For example, studies revealed that siblings of autistic kids display mild developmental delay and mental retardation of unknown etiology or psychopathology, such as affective and anxiety disorders [57]. Other studies show unaffected siblings with an increased rate of dyslexia and specific language impairment [58]. Apart from typical neurodevelopmental deficits, the unaffected siblings are also found to have a higher risk of having lower social responsiveness, delinquent behaviors, and lower receptive/expressive language [58]. Risks of neurodevelopmental and psychiatric disorders among siblings of children with ASD are high [59]. All of these abnormalities can affect their academic performance.

Among the siblings facing a variant of developmental challenges resulting from ASD, we must point out that not all have mental disabilities. Some are subclinical, just like BAP, which means they can still function in society [57]. However, because of the variant deficits, they might suffer from some challenges in their lives, and sub-satisfied academic performance is the easiest way to know as a parent, educator, or physician.

Sibling relationships and their impact on the mental health of unaffected siblings

The sibling relationship is the strongest and longest of all human relationships. Siblings are often children's first playmates, teachers, and role models. Hence, this special relationship affects the development of social, emotional, behavioral, and psychological fields [60]. It is not only important for children with autism spectrum disorder but also for the unaffected siblings. A great sibling relationship and family support provide a better environment for growth, controlling temper, and stabilizing their emotions [61]. In contrast, if kids grow up in a stressful or even resentful sibling relationship and tense family environment, their emotions will become unstable. This may lead to higher possibility of mental disorders [11].

So far, there has been no consistent finding in childhood sibling relationships between autistic kids and their siblings in the past studies [60-64]. In some studies, siblings showed reduced interactions and less closeness and viewed their autistic siblings as a burden [61,62]. In other studies, siblings admired their autistic siblings and were happy with their relationship [63]. There is both positive and negative experience for the siblings when living with their autistic siblings (Table 1)

**Table 1:** The positive and negative experiences of the unaffected siblings when living with autistic patients

The life experience of living with autistic patients for their siblings	
Positive	Negative
Admiration	Burden
Responsible	Double-standard parenting
Advocacy	Overwhelming responsibility
Protection	Self-isolation
Family routine	Embarrassment
Consideration	Stress
Empathy	Meltdowns

The age difference of the samples might cause these two different results shown in Table 1 [63]. Recent studies claim that the symptoms of ASD change with age [64]. As a result, the relationship between autistic kids and their siblings may also change [61]. The severity of impairments of communication, social interaction, and behavior in ASD children is likely to become less over time, even though it remains problematic throughout their life [64]. Therefore, the relationships tend to improve over time, although the impact caused by the impairments of ASD may continue during adolescence and adulthood. Another possibility is that when siblings grow up, they are able to deal with the stress caused by their autistic siblings [61]. Hence, the relationship between the siblings and their autistic brothers and sisters is not concluded [7].

However, one thing is concluded - most siblings of individuals with ASD had relatively limited family interactions [48]. They were more isolated and lonely than siblings of normal kids or children with other disabilities such as Down syndrome [62]. They also complained that "double-standard parenting", the unfair treatment in the family caused by the mothers who were laxer towards the misbehavior of their autistic siblings, is one of the causes [7]. And the unpredictable, aggressive behaviors of their autistic siblings might be another cause.

Mandleco and Webb reviewed studies about the siblings living with kids with mental disorders and found that unaffected siblings of autistic kids had more negative feedback towards their siblings than children with Down syndrome. Unsurprisingly, more siblings found ASD stressful and negatively impacting their interactions with peers [65]. Furthermore, some siblings worry about their future and that of their autistic siblings because they have to take care of them when their parents get old [7].

The relationship between the unaffected siblings and their ASD siblings is complex [61]. Stress, embarrassment, isolation, and worry are common among them. Meanwhile, they also show empathy, care, and protection to their ASD siblings [7]. Now, we will focus on how this relationship and paradoxical emotion affect their mental health and eventually impact their academic performance. Studies showed that siblings interacting with children with ASD for a long term face a significant impact on their mental health, causing depression and anxiety [66]. A meta-analysis of 69 articles concluded that significant impairments across social, emotional, behavioral, and psychological domains were found in unaffected siblings of individuals with ASD [67]. How did this happen? First, deficits in problem-solving skills and sometimes unpredictable behavior were common causes. For example, they complained of difficulties dealing with their sibling's aggression, meltdowns, and social problems [68]. They also experience challenges while interacting with their sibling, especially problematic and unpredictable behavior [23]. Similarly, the emotional burden will grow heavier when they receive negative attitudes and comments while interacting with their classmates and teachers in school [69]. Bendix and Sivberg's research concluded that they would prefer to self-isolate from the rest of the family because of fear of physical violence from their autistic siblings, which significantly impacts their friendships and social life. For instance, some unaffected siblings complained about being uncomfortable inviting friends home. They felt embarrassed to tell peers about their autistic siblings because they thought they needed to explain their siblings' situations [69]. Research also showed that the unaffected siblings are engaged in fewer prosocial behaviors than other community children [68]. Their peer relationships have raised concerns from their parents [60]. The paradoxical relationship with their autistic siblings, the negative emotion obtained from challenging interactions, and the self-isolation from family, will be challenges to the mental health of siblings of autistic kids, especially the kids.

The overwhelming responsibilities also put a significant burden on the unaffected siblings' mental health [48]. Many siblings are worried about taking care of their autistic siblings and protecting their autistic siblings from being bullied or hurting themselves/others. The responsibility is above the expectation of their age [70]. They also need to do extra household duties so their parents can rest [48]. The majority of siblings of autistic children unsurprisingly felt angry, frustrated, upset, hurt, and embarrassed if they got negative attitudes or disapproving comments about their autistic sibling [71].

Apart from that, parents' stress can also negatively impact unaffected siblings psychologically. Studies showed that around 3/4 (78%) of the parents had impaired family functioning and family stress. Consequently, this stress affected the growth of the siblings. Unsurprisingly, parents' subjective quality of life (QoL) was also not contented [72], which inevitably affects the unaffected siblings' mental health. Many siblings reported having differential parenting, less attention from parents, and different expectations from them [48]. They also complained of feeling they needed to give in to avoid further conflict all the time, such as changing their behavior and keeping things to themselves, because they did not want to put more pressure on their parents [7,14]. These experiences weighed heavily on their well-being, with many feeling upset, shame, embarrassed, angry, fearful, and socially isolated [48]. A study investigated academic performance among unaffected siblings of individuals with ASD. Sixty-six youths with a clinical diagnosis of ASD and 132 of their unaffected siblings were recruited, aged eight to nineteen. The result showed that unaffected siblings had a bad attitude toward schoolwork and more severe behavioral problems in class compared to the general population [53].

Limitations and problems that need to be solved

As we mentioned, studies on the siblings of individuals with autism spectrum disorder have just started. The sample size, classification, age group, and demographic factors are still not yet standardized among the currently limited research about academic performance. Randomized controlled trials with larger sample sizes and comparisons based on demographic factors are needed. Whether early intervention before a diagnosis could optimize outcomes for those high-risk siblings with ASD or other developmental challenges is still unknown. Research addressing this issue is currently underway. Different methods were used for the currently existing six intervention group studies, and different standards were applied (including psycho-education about their sibling's disability, opportunities to share experiences, coping skills enhancement, and relaxation techniques) [70]. It is hard to interpret which method is better, and the long-term outcome is still unknown. Methods comparison study should be done to set a standard to guide the intervention.

## Conclusions

Siblings of individuals with autism spectrum disorder (ASD) are at a high risk of recurrent ASD (10-20%), broader autism phenotype (BAP), attention-deficit hyperactivity disorder (ADHD), oppositional defiant disorder (ODD), dyslexia, language delay, and psychological health problems (such as depression and anxiety). All of these can affect their ability to cope with society, and the early sign might be poor academic performance. Due to double-standard parenting, most parents are reported to spend most of their time and energy taking care of children with ASD. They might not realize the subtle signs of the other siblings, especially the younger ones. It will be unsurprising that school teachers are the first ones to notice the sub-clinical signs of their lower academic performance. We try to figure out the possible causes of the relative poor school performance in order to find a way to detect it as early as we can. Early detection and intervention can improve the clinical outcomes of ASD, ADHD, dyslexia, and other neurodevelopmental challenges. Regarding the mental health of the siblings, early intervention can help to develop the proper coping skill to handle the stress they are facing. 

Academic performance is a quantitative standard, easy to be noticed by everybody, and can give us a red flag for its possible underlying causes. Then, more in-depth assessments and interventions should help in improving the outcomes. Also, support groups addressing stress and poor learning skills should be encouraged in every sibling of an autistic patient.
